# Enhanced Mechanical and Acoustic Properties of Basalt Fiber/Polyurethane Composites by Silane Coupling Agents

**DOI:** 10.3390/polym17010061

**Published:** 2024-12-29

**Authors:** Mengchen Ge, Xiaodong Li, Fei Han, Xing Su, Hao Jiang, Youhao Liu, Yangwei Wang, Meishuai Zou

**Affiliations:** 1 School of Materials Science and Engineering, Beijing Institute of Technology, Beijing 100081, China; gmcv587@hotmail.com (M.G.); 6120220003@bit.edu.cn (X.S.); jiangh@bit.edu.cn (H.J.); wangyangwei@bit.edu.cn (Y.W.); 2 Division of Functional Materials Research, Central Iron and Steel Research Institute, Beijing,100081, China; 3 The Key Laboratory of Biomedical Information Engineering of Ministry of Education, School of Life Science and Technology, Xi’an Jiaotong University, Xi’an 710049, China; feihan@xjtu.edu.cn; 4 Bioinspired Engineering and Biomechanics Center (BEBC), Xi’an Jiaotong University, Xi’an 710049, China; 5 Earth-Panda Advanced Magnetic Material Co., Ltd., Hefei 231500, China; liuyouhao@earth-panda.com; 6 National Key Laboratory of Science and Technology on Material under Shock and Impact, Beijing 100081, China

**Keywords:** surface modification, polyurethane elastomer, microphase separation, composites

## Abstract

Modified basalt microfiber-reinforced polyurethane elastomer composites were prepared by a semi-prepolymer method with two different silane coupling agents (KH550 and KH560) in this study. Infrared spectroscopy was used to quantify the degree of microphase separation and analyze the formation of hydrogen bonding in polyurethane. The interfacial surface and the morphology of fibers and composites from tensile fracture were examined by a scanning electron microscope. Further measurements were performed on an electronic universal testing machine for characterizing the mechanical properties of composites. Moreover, the loss factor and transmission loss of composite materials were obtained from dynamic thermomechanical analysis and acoustic impedance tube, respectively. The suitable concentrations in the modification of basalt fibers were established at 1% for KH550 and 1.5% for KH560. The best overall performance was obtained in KH550-BMF/PUE group, as the properties increased by 31% in tensile strength, 37% in elongation at break, and 21% in acoustic insulation.

## 1. Introduction

With the development of science and advancement of technology, aircraft, automobiles, and rail systems have become the predominant modes of transportation for individuals. The vibration and noise produced by vehicles during operation contribute to a decrease in the fatigue life of components and cause physiological discomfort for drivers [1,2]. Damping materials, known as the vibration- and noise-damping material, possess the capability to absorb and dissipate vibration and sound energy, converting it into other forms of energy, such as electrical, thermal, or magnetic energy [3,4,5]. Polyurethane, a promising versatile and synthetic polymer [6,7,8,9,10], has drawn researcher interests in the field of vibration damping and noise reduction for transportation due to its unique microphase separation structure [11,12,13], flexible designability, and tunability. However, one-component polyurethanes face challenges, including a narrow effective damping temperature range and insufficient mechanical or acoustic properties under extreme working conditions. As an excellent matrix material, polyurethane can be incorporated with fillers of different sizes and shapes, such as carbon nanotubes, nano silica, and glass fibers [14,15,16]. The derived polyurethane composites exhibit improved properties, which are favored in applications with high demand for vibration damping and sound proofing [17], especially in transportation and construction fields [18].

In recent times, polyurethane modified with inorganic fillers has garnered considerable attention, particularly the utilization of cost-effective, high-performing, and efficient fillers as replacements for expensive reinforcing materials. In contrast to reinforcing materials like carbon fiber and glass fiber, basalt fiber (BF) is produced by basalt rocks that undergo melting and pulling procedures. Due to its eco-friendliness, exceptional mechanical and thermal properties, fire and chemical resistance, and cost-effectiveness [19,20,21], BF-reinforced polymer has emerged as a promising alternative to traditional composites, displaying significant potential in sectors such as construction, aerospace, automotive, fire protection, and environmental conservation [22,23,24].

However, the inherent smooth and inert surface of BF poses challenges in terms of adhesion with resin matrices, leading to a weaker interfacial bond strength [25]. This deficiency significantly impacts the overall properties of the materials, thereby limiting the full realization of BF’s advantages [26]. Consequently, this hinders the further development and adoption of BF and its composites [27]. Therefore, extensive research into the surface-modification techniques of BF and the enhancement of its interfacial bond strength is imperative for the widespread adoption and application of BF-reinforced polymer [28,29,30].

In order to study the influence of coupling agent-modified basalt microfibers on the composition, phase separation and other microstructures and mechanics, and damping and sound insulation properties of polyurethane elastomer composites, coupling agent solutions of different concentrations were prepared to conduct surface testing on basalt microfibers. Modification: A series of modified basalt microfiber-reinforced polyurethane elastomer samples with different coupling agent concentrations were prepared. FTIR, SEM, mechanical, damping, and acoustic tests were conducted on the modified basalt microfibers and the composite materials prepared to analyze the influence of the coupling agent KH550- or KH560-modified basalt microfibers on the structure and performance of polyurethane elastomer materials and determine the optimal formula to fill the research gap in the field of BF-reinforced polyurethane elastomer materials.

## 2. Materials and Methods

### 2.1. Materials

The materials used in this study were sourced from different suppliers. Specifically, methylene diphenyl diisocyanate (MDI), polyether polyol (330 N-4950), and polytetrahydrofuran glycol (PTMG-2000) were obtained from Wanhua Chemical Group Co., Ltd. (Yantai, China). Milli-Q water and 1,4-butanediol (BDO) were acquired from Shanghai Macklin Biochemical Technology Co., Ltd. (Shanghai, China) and applied as a chemical blowing and chain extender, respectively. Additionally, the supply of the homogenizing agent silicone oil G-580 was entrusted to Dongguan Guangsiyuan Polyurethane Material Co., Ltd. (Dongguan, China).

The catalyst utilized in this study was a mixture of gel catalyst (T-12) and foaming catalyst (A-1) in a precise proportion. A-1 catalyst was purchased from Shanghai Zhengui New Material Technology Co., Ltd. (Shanghai, China) and consisted of a 70% solution of double (dimethylaminoethyl) ether and a 30% solution of dipropylene glycol (DPG). The T-12 catalyst was obtained from Xindian Chemical Materials Co., Ltd. (Shanghai, China), and its main component was dibutyltin dilaurate with an effective substance content of 18%. Silane coupling agents, γ-aminopropyltriethoxysilane (KH550) and γ-glycidoxypropyltrimethoxysilane (KH560), were purchased from Sa’en Chemical Technology Co., Ltd. (Shanghai, China) and Meryer Chemical Technology Co., Ltd. (Shanghai, China), respectively. The basalt fibers, which were purchased from Sichuan Pawoke Mineral Fiber Products Co., Ltd. (Guang’an, China), measured 6 mm in length and 18 µm in diameter. 

It is worth noting that the water and BDO used in this study were analytically pure, while the other chemicals were chemically pure.

### 2.2. Preparation of Polyurethane Composites

Pretreatment of raw materials was needed. The raw materials 330 N, PTMG-2000, and BDO were vacuum-dehydrated at 100~110 °C for 2 h and then cooled to room temperature before use. The polyurethane elastomer matrix was made by the semi-prepolymer method as follows: Firstly, the dehydration reaction of P2000 and MDI was conducted under 80~85 °C for 4 h at isocyanate index (R) = 6.45 to produce the prepolymer (component B). Secondly, component A was mixed with component B at R = 1.05, where component A was composed of P2000, 330N, chain extender, water, foam stabilizer, and catalyst with a certain proportion. The mixture was then stirred in a beaker at 2000 r·min^−1^. Afterwards, the homogeneous mixture was immediately poured into the mold, placed at 70 °C for 15 min, and then allowed to solidify at room temperature for 48 h.

The surface-modified basalt fibers were obtained as follows: The solvents were prepared by mixing absolute ethanol and distilled water in a volume ratio of 9:1. Then, the silane coupling agents were added. The basalt fibers were immersed in a mixed solution of selected silane coupling agents (KH550 or KH560) with a respective mass fraction of 0.5%, 1.0%, 1.5%, and 2%. Afterwards, the mixtures were stirred for 30 min and ultrasonicated for 10 min. The modified basalt fibers were prepared after cleaning and drying.

Polyurethane composite materials were composed of polyurethane elastomer matrix and the incorporated basalt microfibers. In Supplementary Figure S1, SEM images demonstrates the difference seen in basalt fibers before and after ball milling. Basalt fibers were crushed for different periods with a crusher to obtain basalt microfibers with different length-to-diameter ratios; the parameters corresponding to different milling times are presented in Table 1. Based on previous investigation, the ideal filler introduction amount of 6 g of basalt microfibers was introduced for each 100 g of polyurethane matrix (around 5.6 wt% modified basalt fibers addition in composites) to prepare all composite materials in this work. A low-magnification SEM image is provided in the Supplementary Materials. As shown in Supplementary Figure S2, the results indicated that the pretreated basalt fibers were dispersed uniformly in the PUE matrix, and no obvious agglomeration was observed.

### 2.3. Scanning Electron Microscope (SEM)

The S-4800 series (field emission scanning electron microscope), manufactured by Mettler Toledo (Greifensee, Switzerland), was utilized for investigation of the surface alterations of the basalt fibers and the cross-sectional morphologies of the composites. Generally, the composite material samples were sliced into small fragments measuring 5 mm × 5 mm in surface area and coated with a thin gold layer. Subsequently, these specimens were placed within a vacuum sample chamber for SEM morphology analysis.

### 2.4. Fourier Transform-Infrared Analysis (FTIR)

Fourier-transform infrared spectroscopy (Nicolet 6700, Thermo Fisher Scientific, Waltham, MA, USA) was applied to analyze the PUE matrix and modified BMF/PUE composites. The prepared samples were studied in the attenuated total reflection mode with a background of air. The instrument’s resolution was set to 4 cm^−1^, and each sample was measured 32 times over the wavelength range of 450–4000 cm^−1^.

### 2.5. Mechanical Tests

According to established criterion GBT 528-2009 (identically adopts 1S0 37:2005 and lS0 37:2005/Cor1:2008) [31], which describes the determination method of tensile stress–strain properties for rubber, vulcanized or thermoplastic, the mechanical properties of the analyte were verified by CMT4104 series, the universal testing machine from MTS Industrial Systems Co., Ltd. (Shenzhen, Guangdong, China). The average analyte thickness was 2.0 ± 0.2 mm, and the experimental velocity was set to be 500 mm·min^−1^. The sample was cut into a dumbbell-shaped specimen with a test gauge length of 20 ± 0.5 mm and a narrow section width of 5 ± 0.1 mm (Supplementary Figure S3). There were at least 3 samples for one set measurement.

### 2.6. Dynamic Thermomechanical Analyzer

The dynamic thermomechanical analyzer (DMA/SDTA861e, Mettler toledo Co., Ltd., Zurich, Switzerland) with liquid nitrogen refrigeration was used to test the dynamic thermomechanical properties of the prepared polyurethane elastomer matrix and composite material. The shear mode was selected, and the frequency was 10 Hz. The test temperature was within the range of −80~80 °C. The heating rate was set to be 3 °C·min^−1^, and the temperature of the sample was scanned. The sample size for DMA test is 5 × 5 × 1 mm. The maximum thickness should be no larger than 2 mm.

### 2.7. Acoustic Performance Tests

In order to study the acoustic performance of the prepared materials, the sound transmission loss (STL) was recorded using a 100 mm diameter acoustic impedance tube (BK4206T, Brüel & Kjær, Nærum, Denmark) for low-frequency detection from 50 to 1600 Hz. The thickness and diameter of the samples prepared were 2 mm and 100 mm, respectively. The STL tests were carried out with the following experimental settings: a temperature of 16 °C and a relative humidity of 72%.

## 3. Results and Discussion

### 3.1. Characterization of Modified Basalt Microfibers (BMF) and BMF/PUE Composites

**Figure 1:**
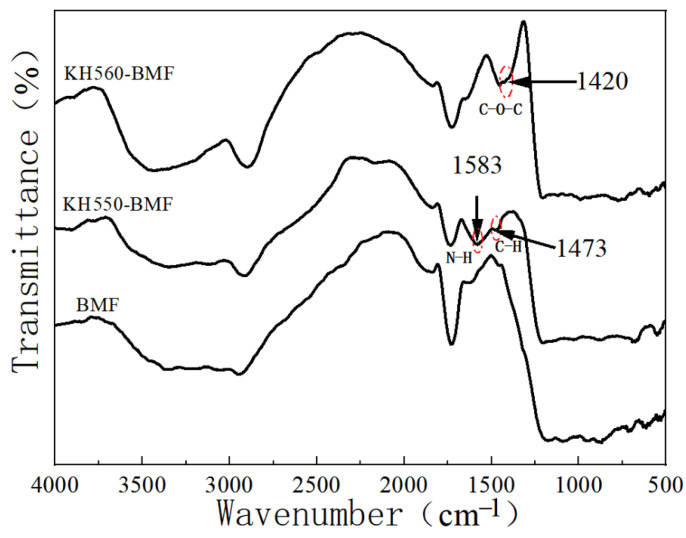
FTIR spectrum of basalt microfibers and modified basalt microfibers.

FTIR was performed on the neat basalt microfibers (BMFs) and surface-modified basalt microfibers (KH550-BMF or KH560-BMF). The obtained spectrum is shown in Figure 1, and the infrared characteristic absorption of the silane coupling agents’ molecules could be observed. Compared to BMF, new bond formations were observed in the KH550-BMF and KH560-BMF. As can be seen in Figure 1, there are absorption peak at 3420 cm^−1^ and 2900 cm^−1^ in the FTIR spectra of all specimens, which can be assigned to the stretching vibration of hydroxy groups. The peak at 1583 cm^−1^ on the KH550-BMF curve could be ascribed to the bending vibration of N-H, while the peak at 1473 cm^−1^ could be attributed to the bending vibration of C-H in KH550 chains. The characteristic absorption peak at 1420 cm^−1^ corresponds to the C-O-C bonds in the epoxy group of KH560. In summary, these typical peaks indicated that the surface modification of basalt microfibers was successful.

**Figure 2:**
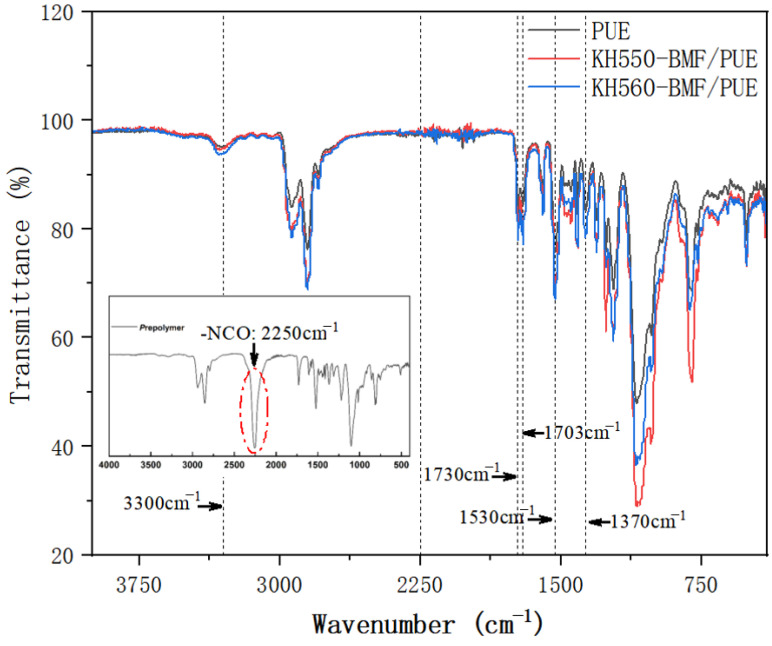
FTIR spectrum of uncoated BMF/PUE and modified BMF/PUE composites.

Figure 2 displayed the FTIR spectrum of uncoated BMF/PUE and modified BMF/PUE composites (KH550-BMF/PUE and KH560-BMF/PUE). The peak at 3300 cm^−1^ was due to N-H stretching vibration. The peaks at 1730 cm^−1^ and 1703 cm^−1^ were characteristic of ester carbonyl. The one at 1530 cm^−1^ was the characteristic absorption peak of C-N-H bending vibration. The peak at 1370 cm^−1^ was attributed to the symmetrical deformation vibration of the methyl groups, and no obvious splitting was seen. This indicated that there was barely branching in the molecular chains. There were no apparent absorption peaks, such as -NCO characteristic absorption peak (see from the inset graph of prepolymer, 2250 cm^−1^). The absence of isocyanate signal indicated that the reaction was fairly complete. Therefore, almost no side reaction was observed within the synthesis of designed composites. 

### 3.2. Phase Structure Analysis

**Figure 3:**
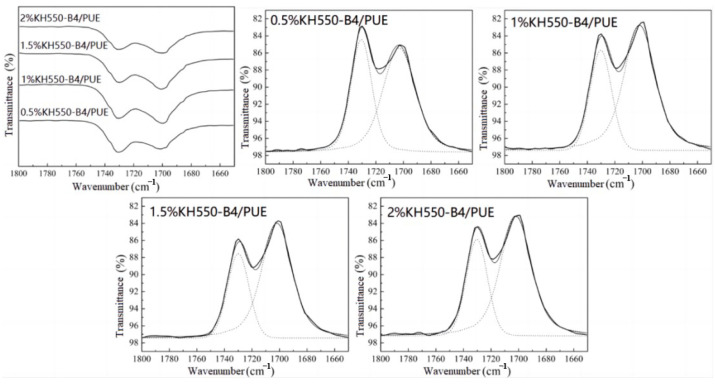
FTIR spectra of the carbonyl part of KH550-B4/PUE composites and Gauss–Lorentz peak splitting results.

**Figure 4:**
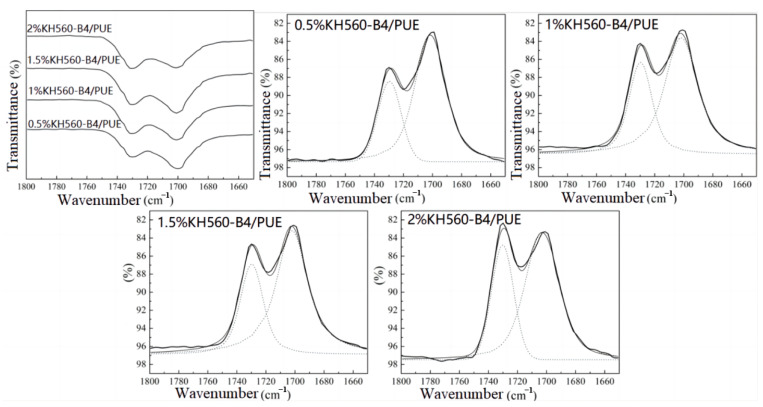
FTIR spectra of the carbonyl part of KH560-B4/PUE composites and Gauss–Lorentz peak splitting results.

The phase structure of polyurethane was closely related to the hydrogen bonding force in the system. Therefore, the analysis of hydrogen bonding in the system was the primary task of studying the microphase separation of polyurethane. In polyurethane, hydrogen bonds formed between the amino groups of carbamate, acting as electron donators, and the highly negative carbonyl groups, acting as electron acceptors. FTIR was a simple and direct method to analyze the hydrogen bonds in polyurethane, as shown in Figure 2. Gauss–Lorentz peak splitting was performed on the wavenumber range of 1800~1650 cm^−1^, which corresponded to the waveband for carbonyl groups of polyurethane. The results are shown in Figure 3 and Figure 4, and the peak splitting data are listed in Table 2. The hydrogen bonding index (HBI) was defined as the ratio of the peak area of the hydrogen-bonded carbonyl group to the peak area of the free carbonyl group, which reflected the degree of hydrogen bonding. 

Figure 3 and Figure 4 show the infrared peak splitting results of the carbonyl part of the KH550-B4/PUE and KH 560-B4/PUE composite materials, and the data are listed in Table 2. As the coupling agent concentration increases, the hydrogen-bonded carbonyl groups and HBI of the composites first increase and then decrease, and they reach the highest value at a concentration of 1 wt% (1.5 wt% for KH560 groups). At this time, the HBI is 2.50 (2.41 for KH560 groups), and the peak area occupied by hydrogen-bonded carbonyl groups reaches 71.43% (70.71% for KH560 groups). The increase in HBI indicates that the number of free carbonyl groups decreases. More carbonyl groups in the molecules form hydrogen bonds. A small amount of coupling agent is attached to the fiber surface. Through the action of the coupling agent, the structure between the molecular chains is more stable, and the hydrogen bonding effect is obvious. It has been documented that the properties of polymers are significantly influenced by hydrogen bonding [32]. The adhesive and mechanical strengths, along with additional characteristics, such as hydrophobicity and corrosion resistance, exhibit enhancement with the escalation of hydrogen bonding [33,34,35,36]. Nevertheless, it is imperative to maintain a controlled level of hydrogen bonding to preserve the requisite equilibrium, as excessive hydrogen bonding may disrupt these properties. In other words, it will hydrolyze itself and cause crosslinking, reducing the micro-phase separation between the fiber and the matrix when the coupling agent concentration is too high.

### 3.3. Mechanical Properties

**Figure 5:**
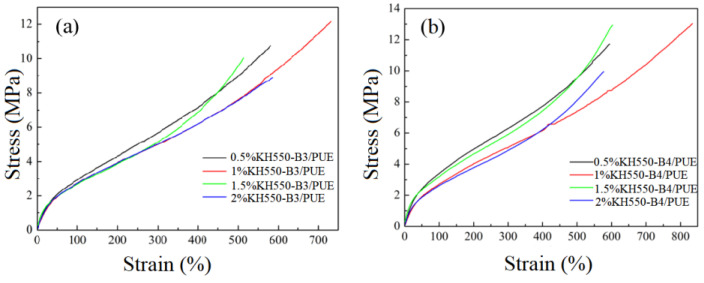
Stress–strain curves of PUE matrix and different composites with (**a**) KH550-B3/PUE and (**b**) KH550-B4/PUE.

**Figure 6:**
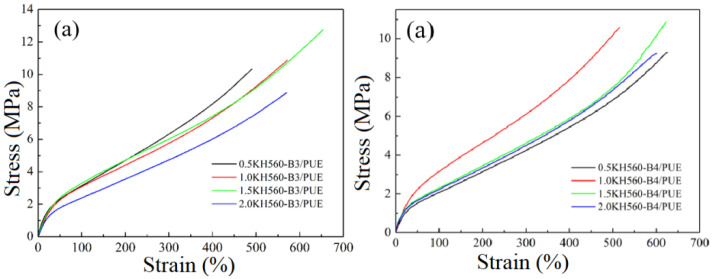
Stress–strain curves of PUE matrix and different composites with (**a**) KH560-B3/PUE and (**b**) KH560-B4/PUE.

In the actual applications, adequate mechanical properties (i.e., elasticity and toughness) of composite materials were vital [37]. Therefore, an important goal in forming BF/PUE composites is to achieve significant enhancements in mechanical properties with BF fillers content of only a few percent. For the tensile properties of these polyurethane composites, typical stress–strain curves of PUE containing different contents of KH550 or KH560 BF/PUE are shown in Figure 5 and Figure 6, where both the tensile strength and the elongation to break of different composites increase substantially. The more detailed mechanical properties of PUE composites at different compositions are given in Table 3 and Table 4. In Figure 5, all curves show typical rubber-like mechanical behaviors, which underwent ductile fracture without yielding. The composites in Figure 5 were prepared by incorporating KH550-B3 or KH550-B4 with polyurethane, while the composites in Figure 6 were prepared by introducing KH560-B3 or KH560-B4 fillers. It can be found from the figure and table that the tensile properties of the composite material first increase and then decrease as the amount of coupling agent KH550 increases, reaching the highest when KH550 is 1.0 wt%. At this time, for the sample 1.0% KH550-B4/PUE, the tensile strength (13 MPa) and elongation at break (835%) are the largest, which are increased by 31% and 37%, respectively, compared with the matrix. Compared with the reported modified basalt fiber polyurethane composites [38], the composites prepared in this study not only show superior mechanical properties (13 MPa vs. 8.8 MPa in tensile strength and 835% vs. 329% in elongation at break) but also consumed less basalt fibers (5.6 wt% vs. 20~30 wt%), indicating good cost-effectiveness and broad prospective in future applications, especially in automotive damping materials. The modules of elasticity vary in the same trend with tensile strength and elongation at break, with around 21% enhancement observed in comparison with PUE matrix. Overall, the KH550 coupling agent at a concentration of 1.0 wt% has the most obvious modification effect on basalt fibers, while the strength of fiber composite polyurethane materials of different sizes does not change greatly. It can be seen that the presence of the coupling agent can balance part of the effect of fibers size. 

Figure 6 shows the mechanical properties of KH560-B3/PUE composite samples, and the corresponding data are listed in Table 4. It can be found from the figure and table that as the coupling agent concentration increases, the tensile strength and elongation at break of the composite material The rate and tear strength first increased and then decreased, reaching the highest when the coupling agent concentration was 1.5 wt%. At this time, the material properties were compared with the polyurethane base slightly lifted. When it comes to B4 groups, the tensile and tear properties of composite materials change to a certain extent as the coupling agent concentration increases, but they are related to the strength of the polyurethane matrix with a tiny difference. Compared with KH550-BMF/PUE, the mechanical properties of KH560-BMF/PUE are generally reduced. It can be seen that, for the coupling agent KH560 and the matrix, the bonding is not as good as that of coupling agent KH550, which may be due to the reaction between the hydroxyl group formed after the hydrolysis of the epoxy group, and the isocyanate may not be as reactive as -NH_2_ and isocyanate groups.

The unmodified BF surface is smooth and lacks active functional groups, thus limiting its application due to poor adhesion with PUE matrix. As can be seen from the data in Supplementary Table S1, the addition of unmodified basalt fibers enhances the tensile strength with decreased elongation at break. In this study, through coupling modification, the bifunctional molecular structure of the coupling agent allows one end to react chemically or physically with the hydroxyl groups on the fiber surface, while the other end with NH_2_ groups or epoxy groups can react chemically with the isocyanate groups of the polyurethane molecules, thereby improving the interfacial compatibility between inorganic fillers and organic matrices. According to the data from Table 2, Table 3 and Table 4, it can be seen that the mechanical properties and their HBI index of the material are correlated and varied in the same trend. Therefore, the formation of hydrogen bonding reinforces the composite material and improves the microphase separation, allowing for more deformation under the same load conditions, ultimately leading to a dual enhancement of tensile strength and elongation at break.

### 3.4. DMA Results Analysis

**Figure 7:**
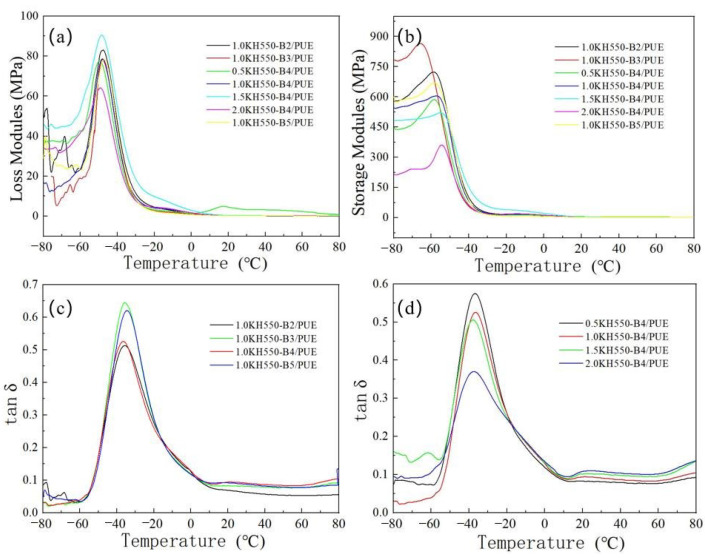
Loss modulus, storage modulus, and loss factor curve of KH550 BMF/PUE composites: (**a**) loss modulus, (**b**) storage modulus, and (**c**,**d**) loss factor of different KH550 BMF/PUE composites.

**Figure 8:**
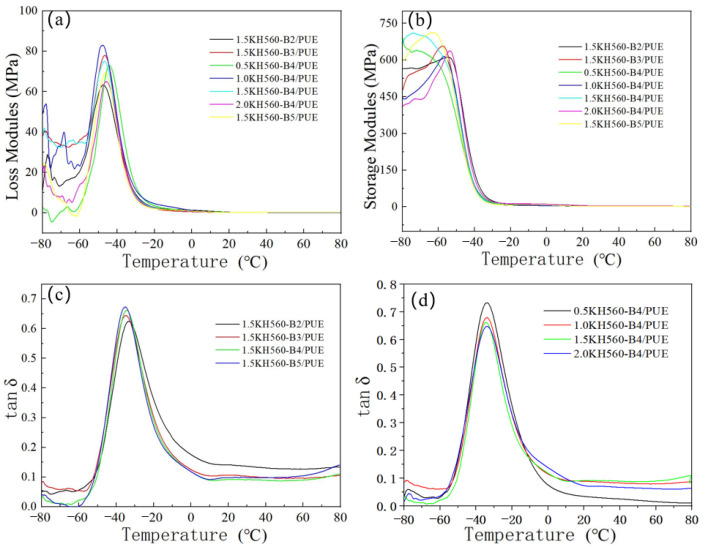
Loss modulus, storage modulus, and loss factor curve of KH560 BMF/PUE composites: (**a**) loss modulus, (**b**) storage modulus, and (**c**,**d**) loss factor of different KH560 BMF/PUE composites.

From the DMA results (Figure 7 and Figure 8), it can be seen that the loss modulus trend of KH550- or KH560-modified basalt fiber-reinforced polyurethane composites first increases and then decreases, reaching a maximum value around −45.75 °C. The trend of storage modulus is similar to that of loss modulus. As the amount of coupling agent added increases, the peak value of the loss factor (tan δ) diminishes (Table 5 and Table 6). Nevertheless, an analysis of the glass transition temperature (T_g_) of the material reveals that variations in coupling agent (KH550 or KH560) concentration have a minimal impact on the T_g_ of the composite material, primarily due to the low concentration of the coupling agent, which remains constant. The structural proportion of soft and hard segments in PUE exerts a negligible influence on the damping characteristics in the vicinity of the glass transition temperature. Conversely, when temperatures surpass 10 °C, the internal segments of PUE progressively commence movement within the composite under shear forces. The intermolecular forces impede the motion of chain segments, resulting in a lag between strain and stress variations. An augmentation in the coupling agent adhered to the basalt fiber surface fosters additional fiber connections to molecular chain segments, thereby enhancing their involvement in internal chain segment motion. This further amplifies the internal friction and enhances the damping effect.

### 3.5. Sound Insulation Performance

**Figure 9:**
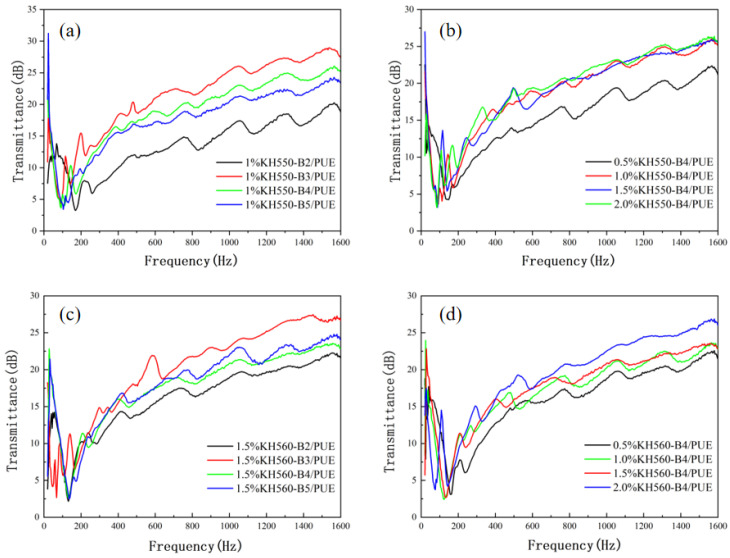
Transmission loss curves from 0 to 1600 Hz for different modified BMF/PUE composites: (**a**) 1% KH550 with different ball milling time, (**b**) same milling time with different KH550 content, (**c**) 1% KH560 with different ball milling time, and (**d**) same milling time with different KH560 content.

The acoustic performance of composites could be expressed by transmission loss. In this study, the amount of sound insulation obtained by the standing wave method was shown in Figure 9. The sound insulation curve of the KH550 coupling agent-modified BMFs/PUE composite material is shown in Figure 9a,b. In Figure 9a, the best sound insulation effect is B3/PUE, followed by B4/PUE and B5/PUE; sample B2/PUE has the lowest sound insulation effect. From the sound insulation curves of the second set of composite materials, it can be seen that the coupling agent has the lowest sound insulation when the concentration is 0.5 wt%. The sound insulation curves basically coincide when the coupling agent concentration is 1.0 wt%, 1.5 wt%, and 2.0 wt%, which shows that when the coupling agent concentration reaches 1.0 wt%, the sound insulation effect of the composite material has entered a plateau stage. At this stage, the increase in coupling agent concentration has little effect on the sound insulation performance of the sample. The relationship between sound insulation and fiber size reveals that composite materials prepared from B3 fiber have the highest performance.

The sound insulation curve of the KH560 coupling agent-modified BMFs/PUE composite is shown in Figure 9c,d. It can be seen that the sound insulation of the sample B3/PUE composite is the highest, and the B2/PUE composite material has the lowest sound insulation, indicating that the larger the size of the filler, the easier it is to produce defects in the matrix. The presence of pores will allow sound waves to directly transmit through the material, failing to block the propagation of sound waves. The composite material made of fibers with B3 size length has the highest sound insulation. It can be seen that the presence of basalt fiber can improve the sound insulation performance of the material. The sound insulation effect at medium and high frequencies is better than that at low frequencies (below 400 Hz), and the maximum can reach 27.28 dB, around 5 dB higher than the performance of the PUE matrix. Figure 9d shows the sound insulation curves of the sample B4/PUE composites. It can be seen that the sound insulation of the composite material increases with the increase in coupling agent concentration. Unlike the modification effect of coupling agent KH550, coupling agent KH560 keeps improving when the concentration reaches 2.0 wt%.

### 3.6. Surface and Composite Tensile Fracture Interfacial Morphology

**Figure 10:**
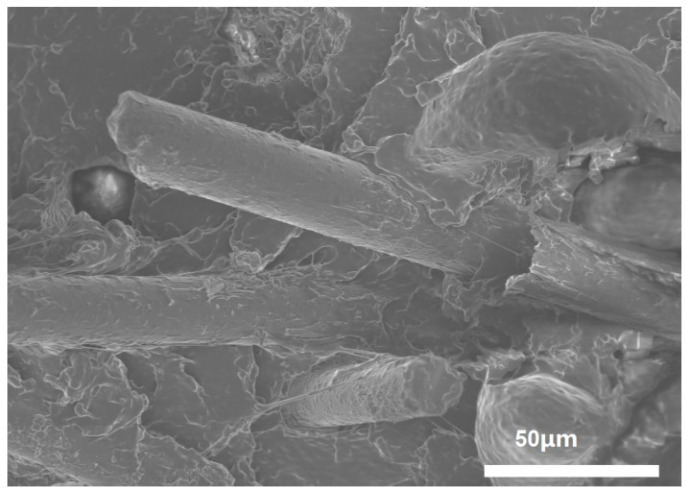
Cross-sectional SEM images of 1 wt%-KH550-B4/PUE.

The best mechanical properties were found for 1.0 wt%KH550-B4/PUE. SEM analysis of its tensile section is shown in Figure 10. It can be seen that the fibers are interspersed in the polyurethane matrix, and the fibers and the matrix are tightly bonded and have evident bonding. Upon exposure to external forces, composite materials leverage high-strength, high-modulus basalt fibers as their structural skeleton, which endures a portion of the load. As these fibers debond and are extracted from the polyurethane matrix, the interaction between basalt fibers and the PUE matrix induces them to carry an additional portion of the load, thereby enhancing the mechanical properties of the composite. Furthermore, chemical modifications with KH550 or KH560 generated a stronger chemical bond between the fibers and the PUE matrix, leading to improved interfacial adhesion.

**Figure 11:**
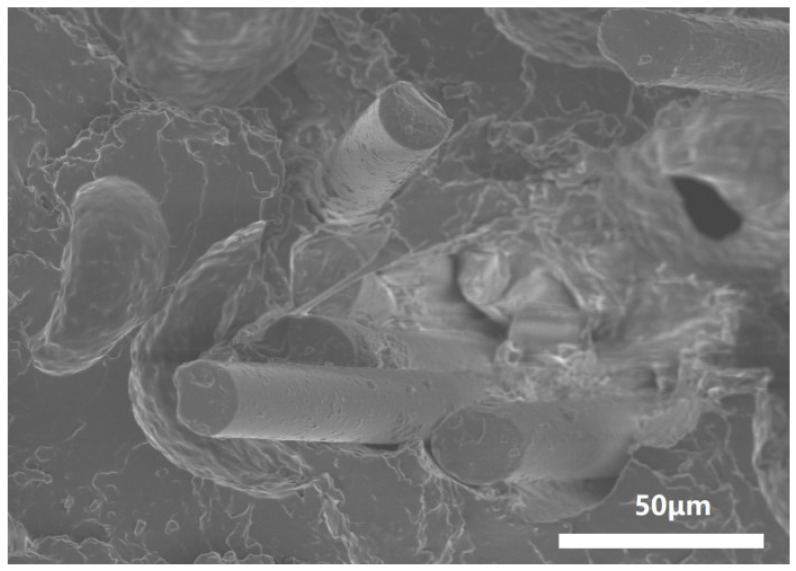
Cross-sectional SEM images of 1.5 wt%-KH560-B4/PUE.

**Figure 12:**
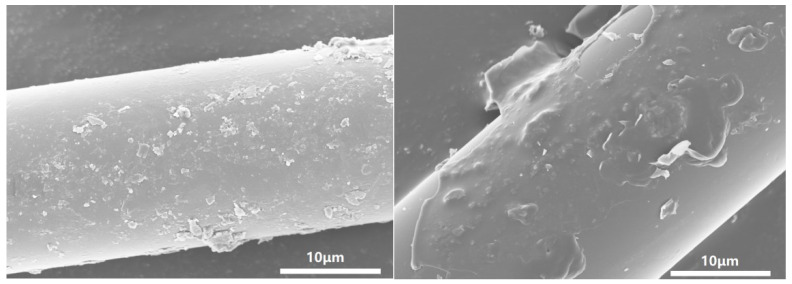
SEM images of KH550 (**left**) and KH560 (**right**) surface-modified basalt fibers.

Sample 1.5 wt%KH560-B4/PUE was selected, and its tensile section was analyzed by SEM. As shown in Figure 11, it can be seen that fibers are interwoven within the polyurethane matrix, with some fibers being closely bonded to the matrix, and the friction between them plays a positive role in the mechanical properties. However, some closely bonded fibers are observed with the increased content of KH560. There are obvious gaps between them and the matrix, thus reducing the mechanical properties of the composite material. In comparison, the interfacial bonding between fiber and polyurethane matrix after modification by coupling agent KH560 is not as good as that after modification by coupling agent KH550. This can be further confirmed in SEM images of KH550- and KH560-modified basalt fibers. The surface morphology of the basalt microfibers after soaking in the coupling agent was observed by SEM, and the electron microscopy pattern was obtained as shown in Figure 12. It can be seen that there are evenly distributed and raised small particles on the surface of KH550-BMF, while a film is formed on the fiber surface of KH560-BMF to wrap the fibers and particles. In comparison, KH550-modified BF has higher surface roughness than KH560-modified BF, which may be one of the reasons for the performance difference between the two.

## 4. Conclusions

This study utilized basalt microfibers as reinforcing materials and polyurethane elastomer as matrix materials to prepare BMF/PUE composites using the semi-prepolymer method. The basalt fibers were surface-modified using the silane coupling agents KH550 or KH560 for composites preparation and further investigation. The type and concentration of the agent affect the hydrogen bond structure of the molecular chain, degree of phase separation, mechanical properties, damping properties, and sound insulation properties of the composite material. 

The degree of carbonyl hydrogen bonding and phase separation of fiber-reinforced polyurethane composites modified by coupling agent have a certain correlation with the coupling-agent concentration. The optimal concentrations for the modification of basalt fibers with KH550 and KH560 were established at 1% (the degree of hydrogen bonding of carbonyl groups is 71.43%, HBI = 2.50) and 1.5% (the degree of hydrogen bonding of carbonyl groups accounts for 70.71%, HBI = 2.41), respectively. The mechanical and acoustic test results show that the composite material (1.0KH550-B4/PUE) prepared by using 1 wt% KH550 coupling agent to modify the basalt microfibers has the best overall performance. The tensile strength was 13 MPa, and the elongation at break was 835%, which were 31% and 37% higher than that of the matrix, respectively. The degree of hydrogen bonding (71.43%) and the degree of microphase separation (HBI = 2.50) of this sample were relatively high, the maximum damping factor moves to the high temperature direction, and the sound insulation reaches 27 dB. The differences in performance could be attributed to the surface roughness and surface activity of the modified basalt fibers. The purpose of coupling treatment on basalt fiber fillers in this study was to improve the interface between the filler and the matrix. The coupling agent acts as a bridge connecting the polyurethane matrix and the basalt fibers. Compared to PUE matrix, the prepared modified basalt fiber polyurethane composites showed over 30% enhancements in mechanical properties and over 20% increasement in acoustic properties. There is potential for future applications in vibration- and noise-reduction materials for transportation or construction. At present, the research results have not fully realized the performance capabilities. Future work will involve grafting carbon nanotubes or dopamine modification to increase the crosslinking points between the filler and matrix, enhance hydrogen bonding, and prepare multi-scale fiber reinforcements. This will expand the original two-dimensional coating structure into a three-dimensional network structure, further strengthening the performance of the filler-reinforced polyurethane composites.

## Figures and Tables

**Table 1:** The parameters corresponding to different milling times.

Size of Chopped Basalt Fiber	Name and Number	Milling Time/min	Length After Milling/µm	Aspect Ratio (L/D, Length to Diameter)
6 mm × 18 µm	B2	2	75–200	4.2–11.1
	B3	3	50–100	2.8–5.6
	B4	4	25–75	1.4–4.2
	B5	5	<50	<2.8

Note: B refers to the abbreviation of basalt microfibers (BMFs), and the number next to the letter indicates the ball-milling time.

**Table 2:** Gauss–Lorentz peak splitting results and HBI of carbonyl groups.

Sample	Type	Bonded -C=O	Free -C=O	HBI
PUE	Peak (cm^−1^)	1700	1730	1.36
	Area (%)	57.70	42.30	
0.5%KH550-B4/PUE	Peak (cm^−1^)	1703	1730	1.46
	Area (%)	59.29	40.71	
1%KH550-B4/PUE	Peak (cm^−1^)	1702	1730	2.5
	Area (%)	71.43	28.57	
1.5%KH550-B4/PUE	Peak (cm^−1^)	1702	1730	2.15
	Area (%)	67.17	32.83	
2%KH550-B4/PUE	Peak (cm^−1^)	1702	1730	1.98
	Area (%)	66.51	33.49	
0.5%KH560-B4/PUE	Peak (cm^−1^)	1702	1730	1.86
	Area (%)	65.05	34.94	
1%KH560-B4/PUE	Peak (cm^−1^)	1702	1730	2.00
	Area (%)	66.67	33.33	
1.5%KH560-B4/PUE	Peak (cm^−1^)	1702	1730	2.41
	Area (%)	70.71	29.29	
2%KH560-B4/PUE	Peak (cm^−1^)	1703	1730	1.93
	Peak (cm^−1^)	65.9	34.1	

**Table 3:** Mechanical properties of PUE matrix and KH550-BMF/PUE composites.

Sample	Tensile Strength (MPa)	Elongation at Break (MPa)	Modulus of Elasticity (MPa)
PUE matrix	9.9 ± 0.3	608 ± 9	1.20 ± 0.05
0.5%KH550-B3/PUE	10.8 ± 0.2	580 ± 5	1.37 ± 0.03
1%KH550-B3/PUE	12.1 ± 0.4	717 ± 14	1.43 ± 0.08
1.5%KH550-B3/PUE	10.2 ± 1.3	513 ± 24	1.28 ± 0.05
2%KH550-B3/PUE	8.6 ± 0.3	586 ± 10	1.17 ± 0.04
0.5%KH550-B4/PUE	11.7 ± 0.4	590 ± 4	1.37 ± 0.02
1%KH550-B4/PUE	13.0 ± 0.3	835 ± 13	1.45 ± 0.06
1.5%KH550-B4/PUE	12.3 ± 0.7	592 ± 12	1.38 ± 0.03
2%KH550-B4/PUE	9.6 ± 0.5	565 ± 13	1.18 ± 0.02

**Table 4:** Mechanical properties of same-content HNT/PUE with different preparation.

Sample	Tensile Strength (MPa)	Elongation at Break (MPa)	Modulus of Elasticity (MPa)
PUE matrix	9.9 ± 0.3	608 ± 9	1.20 ± 0.05
0.5%KH560-B3/PUE	10.3 ± 0.4	491 ± 8	1.58 ± 0.02
1%KH560-B3/PUE	10.9 ± 0.3	572 ± 8	1.56 ± 0.03
1.5%KH560-B3/PUE	12.4 ± 0.5	643 ± 13	1.62 ± 0.02
2%KH560-B3/PUE	8.9 ± 0.4	570 ± 9	1.53 ± 0.02
0.5%KH560-B4/PUE	9.0 ± 0.2	618 ± 9	1.47 ± 0.03
1%KH560-B4/PUE	10.6 ± 0.3	516 ± 14	1.48 ± 0.04
1.5%KH560-B4/PUE	10.9 ± 0.4	623 ± 12	1.61 ± 0.03
2%KH550-B4/PUE	9.3 ± 0.4	601 ± 11	1.47 ± 0.05

**Table 5:** The maximum damping factor, corresponding temperature, and effective damping temperature range for KH550 BMF/PUE composites.

Sample	tan δ Max	T tan δ Max (°C)	tan δ Max ≥ 0.3 (°C)
PUE	0.65	−39.85	−51.35~−24.06
1.0KH550-B2/PUE	0.51	−35.55	−46.22~−21.87
1.0KH550-B3/PUE	0.65	−36.42	−46.66~−21.00
0.5KH550-B4/PUE	0.57	−36.72	−48.03~−22.08
1.0KH550-B4/PUE	0.53	−36.49	−46.42~−22.51
1.5KH550-B4/PUE	0.51	−37.63	−48.03~−23.18
2.0KH550-B4/PUE	0.37	−37.16	−45.12~−27.29
1.0KH550-B5/PUE	0.62	−35.75	−45.79~−20.13

**Table 6:** The maximum damping factor, corresponding temperature, and effective damping temperature range for KH560 BMF/PUE composites.

Sample	tan δ Max	T tan δ Max (°C)	tan δ Max ≥ 0.3 (°C)
PUE	0.65	−39.85	−51.35~−24.06
1.5KH560-B2/PUE	0.62	−33.98	−44.01~−16.19
1.5KH560-B3/PUE	0.66	−33.98	−45.62~18.63
0.5KH560-B4/PUE	0.73	−33 11	−46.22~17.52
1.0KH560-B4/PUE	0.68	−33.97	−45.79~−18.83
1.5KH560-B4/PUE	0.66	−34.41	−44.44~−20.10
2.0KH560-B4/PUE	0.65	−33.98	−46.22~18.39
1.5KH560-B5/PUE	0.67	−35.32	−45.75~−21.40

## Data Availability

Data are contained within the article.

## Supplementary Materials

The following supporting information can be downloaded at https://www.mdpi.com/article/10.3390/polym17010061/s1, Figure S1: SEM images of basalt fibers (a) before crushing and (b) after crushing; Figure S2: The SEM image of dispersed basalt fibers (1% KH550 modified) in PUE matrix; Figure S3: The dumbbell specimen and nominal dimensions (mm); Table S1: Mechanical properties of same filler content composites with different ball milling time.

## References

[1] Zhu G.L., Han D., Yuan Y., Chen F., Fu Q. (2018). Improving damping properties and thermal stability of epoxy/polyurethane grafted copolymer by adding glycidyl POSS. Chinese J. Polym. Sci..

[2] Zheng N., Wang Q.Z., Cui C.X., Yin F.X., Jiao Z.X., Li H.Z. (2020). Fabrication and damping behaviors of novel polyurethane/TiNiCu composites. Physica B..

[3] Wang F.L., Zou L., Zhang Q. (2020). Research progress on modification of polyurethane damping materials. Polyurethane Industry.

[4] Bozkurt Ö.Y., Gökdemir M.E. (2018). Effect of basalt fiber hybridization on the vibration-damping behavior of carbon fiber/epoxy composites. Polym. Compos..

[5] Chiter A. (2021). Rubber’s dissipated energy quantification used in vibratory insulation and protection systems. J. Appl. Polym. Sci..

[6] Bezrodna T.V., Ishchenko A.A., Bezrodnyi V.I., Negriyko A.M., Kosyanchuk L.F., Antonenko O.I., Brovko O.O. (2021). Covalent bonding effects on spectral, photophysical and generation properties of indocarbocyanine dyes in polyurethanes. Opt. Laser. Technol..

[7] Engels H.W., Pirkl H.G., Albers R., Albach R.W., Krause J., Hoffmann A., Casselmann H., Dormish J. (2013). Polyurethanes: Versatile Materials and Sustainable Problem Solvers for Today’s Challenges. Angew. Chem. Int. Ed..

[8] Bezrodnyi V.I., Ishchenko A.A. (2014). High efficiency lasing of a dye-doped polymer laser with 1.06 μm pumping. Appl. Phys. B..

[9] Qian Y.Q., Lindsay C.I., Macosko C., Stein A. (2021). Synthesis and Properties of Vermiculite-Reinforced Polyurethane Nanocomposites. ACS Appl. Mater. Inter..

[10] Misztalewska-Turkowicz I., Coutelier O., Destarac M. (2020). Two Pathways of Thiolactone Incorporation into Polyurethanes and Their One-Pot Double Postfunctionalization. Macromolecules.

[11] Wang Z., Zhang T.F., Zhang Z.J., Ge Z., Luo Y.J. (2016). Effect of hard-segment content on rheological properties of glycidyl azide polyol-based energetic thermoplastic polyurethane elastomers. Polym. Bull..

[12] Zhang H.T., Zhang F., Wu Y.X. (2020). Robust stretchable thermoplastic polyurethanes with long soft segments and steric semisymmetric hard segments. Ind. Eng. Chem. Res..

[13] Krishna G.G., Sourav M., Sutapa S.C., Pintu S., Evgenii K., Chitrangada D.M., Sergei V.K., Priyadarsi D. (2020). Self-Assembly of Amphiphilic Copolymers with Sequence-Controlled Alternating Hydrophilic–Hydrophobic Pendant Side Chains. ACS Appl. Polym. Mater..

[14] Shamsi R., Mahyari M., Koosha M. (2017). Synthesis of CNT-polyurethane nanocomposites using ester-based polyols with different molecular structure: Mechanical, thermal, and electrical properties. J. Appl. Polym. Sci..

[15] Yuan Y.H., Peng C., Chen D., Wu Z.J., Li S.C., Sun T., Liu X. (2012). Synthesis of a coupling agent containing polyurethane chain and its influence on improving the dispersion of SiO2 nanoparticles in epoxy/amine thermoset. Compos. Part A-Appl. S..

[16] da Costa Mattos H.S., Reis J.M.L., Paim L.M., da Silva M.L., Amorim F.C., Perrut V.A. (2014). Analysis of a glass fibre reinforced polyurethane composite repair system for corroded pipelines at elevated temperatures. Compos. Struct..

[17] Praveen S., Bahadur J., Yadav R., Billa S., Umasankar Patro T., Rath S.K., Ratna D., Patri M. (2020). Tunable viscoelastic and vibration damping properties of a segmented polyurethane synergistically reinforced with carbon black and anisotropic additives. Appl. Acoust..

[18] Li X.R., Li J., Wang Y.J., Yuan J., Jiang F., Yu X.Y., Xiao F.P. (2021). Recent applications and developments of Polyurethane materials in pavement engineering. Constr. Build. Mater..

[19] Lopresto V., Leone C., De Iorio I. (2011). Mechanical characterization of basalt fibre reinforced plastic. Compos. Part B-Eng..

[20] Wei B., Cao H.L., Song S.H. (2011). Degradation of basalt fibre and glass fibre/epoxy resin composites in seawater. Corros. Sci..

[21] Borhan T.M. (2012). Properties of glass concrete reinforced with short basalt fiber. Mater. Des..

[22] Zhu H., Wu G., Zhang L., Zhang J., Hui D. (2014). Experimental study on the fire resistance of RC beams strengthened with near-surface-mounted high-Tg BFRP bars. Compos. Part B—Eng..

[23] Wu G., Wu Z.S., Luo Y.B., Sun Z.Y., Hu X.Q. (2010). Mechanical properties of steel-FRP composite bar under uniaxial and cyclic tensile loads. J. Mater. Civil Eng..

[24] Dhand V., Mittal G., Rhee K.Y., Park S.J., Hui D. (2015). A short review on basalt fiber reinforced polymer composites. Compos. Part B—Eng..

[25] Liu H., Yu Y., Liu Y., Zhang M., Li L., Ma L., Sun Y., Wang W. (2022). A review on basalt fiber composites and their applications in clean energy sector and power grids. Polymers.

[26] Pan G.X., Xu J.L., Ren S.B., Chen Z., Zhou K.H. (2024). Study on the epoxy bonding properties of polyphenylene sulfide matrix composites reinforced by montmorillonite and basalt fiber. J. Adhes. Sci. Technol..

[27] Chen Z.W., Huang Y.D. (2016). Mechanical and interfacial properties of bare basalt fiber. J. Adhes. Sci. Technol..

[28] Sun H.M., Xiang D., Zhang J., Tian W., Harkin-Jones E., Wang J.J., Wang M.H., Wang B., Zhao C.X., Li H. (2023). Electrical, mechanical and damage self-sensing properties of basalt fiber reinforced polymer composites modified by electrophoretic deposition. Prog. Nat. Sci.-Mater. Int..

[29] Tang L., Zhang J.L., Tang Y.S., Kong J., Liu T.X., Gu J.W. (2021). Polymer matrix wave-transparent composites: A review. J. Mater. Sci. Technol..

[30] Liu H.Y., Fan X.B., Pan B.L., Zhou Y.X., Zhang L.L., Li M.H., Li Z.Y., Pang X.C. (2024). Simultaneously enhancing tribological and mechanical properties of epoxy composites using basalt fiber/reduced graphene oxide/paraffin wax. Polym. Compos..

[31] Rubber, Vulcanized or Thermoplastic—Determination of Tensile Stress-Strain Properties.

[32] Saha J.K., Rahman M.M., Haq M.B., Al Shehri D.A., Jang J. (2022). Theoretical and Experimental Studies of Hydrogen Bonded Dihydroxybenzene Isomers Polyurethane Adhesive Material. Polymers.

[33] Christopher G., Kulandainathan M.A., Harichandran G. (2015). Comparative study of effect of corrosion on mild steel with waterborne polyurethane dispersion containing graphene oxide versus carbon black nanocomposites. Prog. Org. Coat..

[34] Rahman M.M., Kim H.-D., Lee W.-K. (2009). Properties of Waterborne Polyurethane Adhesives: Effect of Chain Extender and Polyol Content. J. Adhes. Sci. Technol..

[35] Rahman M.M., Kim H.-D. (2006). Synthesis and characterization of waterborne polyurethane adhesives containing different amount of ionic groups (I). J. Appl. Polym. Sci..

[36] Wang Y.-Y., Wyman C.E., Cai C.M., Ragauskas A.J. (2019). Lignin-Based Polyurethanes from Unmodified Kraft Lignin Fractionated by Sequential Precipitation. ACS Appl. Polym. Mater..

[37] Cooper S.L., Tobolsky A.V. (1966). Viscoelastic Behavior of Segmented Elastomers. Text. Res. J..

[38] Jing F.D., Han S., Ge J., Qkin Y., Wang N., Guan Y. (2022). Preparation and properties of modified basalt fiber/polyurethane damping material. Fine Chem..

