# Supplementary material for: Enhanced Mechanical and Acoustic Properties of Basalt Fiber/Polyurethane Composites by Silane Coupling Agents
Source: Polymers (Basel). 2024 Dec 29;17(1):61. doi: 10.3390/polym17010061 (PMC11723291; doi:10.3390/polym17010061)
Supplement: Supplementary file 1 [file polymers-17-00061-s001.zip › polymers-3259710-supplementary.pdf]

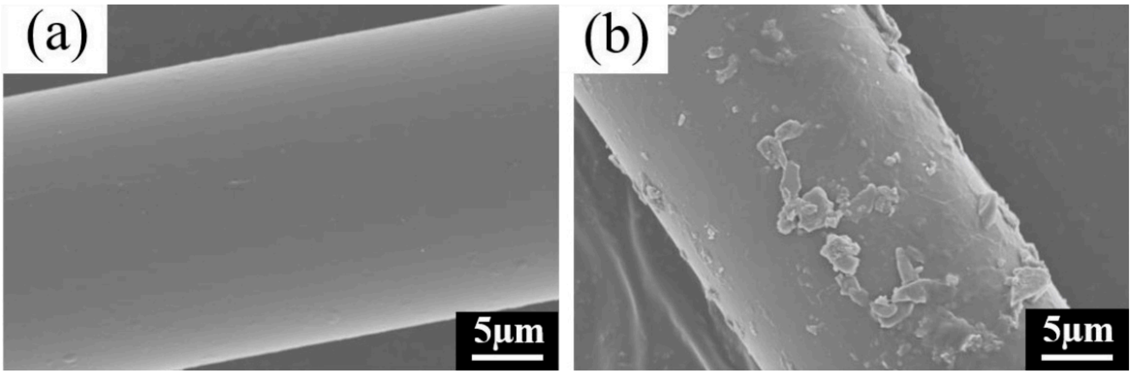

Figure S1. SEM images of basalt fibers (a)before crushing and (b)after crushing

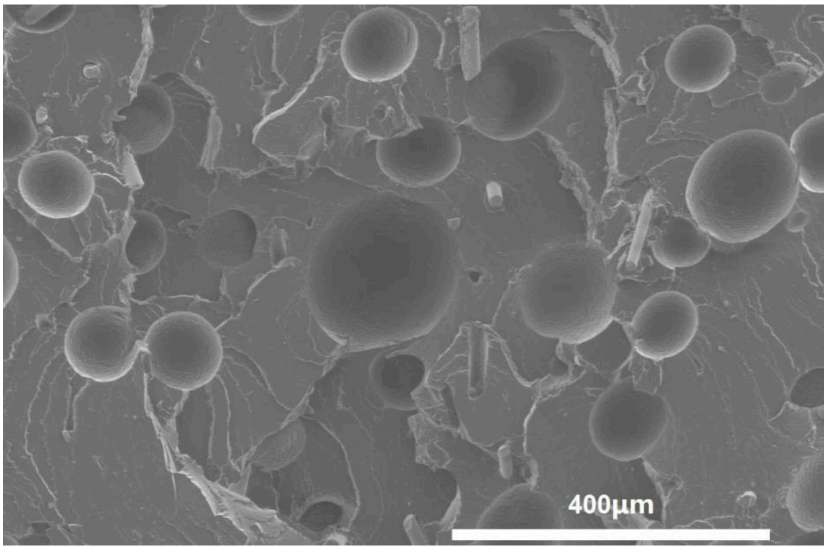

Figure S2. The SEM image of dispersed basalt fibers (1% KH550 modified) in PUE matrix.

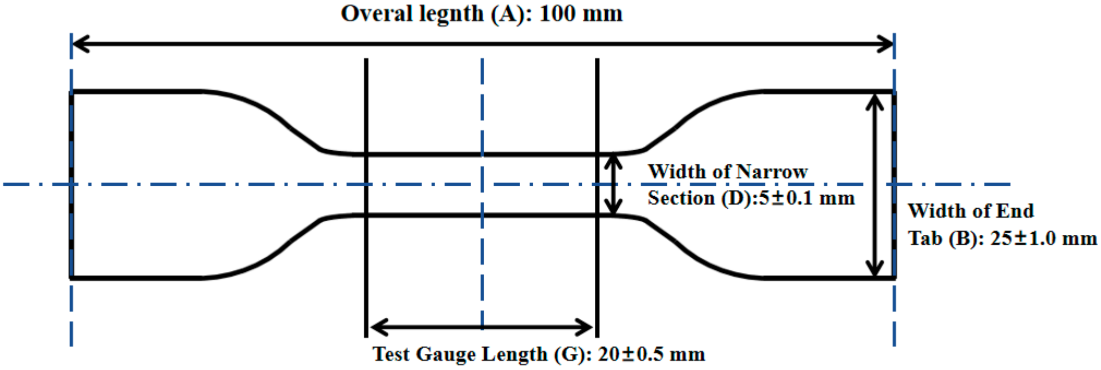

Figure S3. The dumbbell specimen and nominal dimensions (mm)

Table S1. Mechanical properties of same filler content composites with different ball milling time

Sample	Tensile strength(MPa)	Elongation at break(MPa)
PUE matrix	9.9 ± 0.3	608 ± 9
B2/PUE	*9.94* ± 0.4	*518* ± 9
B3/PUE	*11.4* ± 0.6	*569* ± 12
B4/PUE	*10.5* ± 0.4	*554* ± 11
B5/PUE	*10.1* ± 0.3	*540* ± 9
